# Micro-doppler radar to evaluate risk for musculoskeletal injury: Protocol for a case-control study with gold standard comparison

**DOI:** 10.1371/journal.pone.0292675

**Published:** 2023-10-10

**Authors:** Bilal Abou Al Ardat, Jennifer Nyland, Robert Creath, Terrence Murphy, Ram Narayanan, Cayce Onks

**Affiliations:** 1 Pennsylvania State University College of Medicine, Hershey, PA, United States of America; 2 Department of Neural and Behavioral Sciences, Penn State College of Medicine, Hershey, PA, United States of America; 3 Exercise Science Department, Lebanon Valley College, Annville, PA, United States of America; 4 Pennsylvania State University College of Engineering, The Pennsylvania State University, University Park, PA, United States of America; The University of British Columbia, CANADA

## Abstract

**Background:**

Beyond causing significant morbidity and cost, musculoskeletal injuries (MSKI) are among the most common reasons for primary care visits. A validated injury risk assessment tool for MSKI is conspicuously absent from current care. While motion capture (MC) systems are the current gold standard for assessing human motion, their disadvantages include large size, non-portability, high cost, and limited spatial resolution. As an alternative we introduce the Micro Doppler Radar (MDR); in contrast with MC, it is small, portable, inexpensive, and has superior spatial resolution capabilities. While Phase 1 testing has confirmed that MDR can identify individuals at high risk for MSKI, Phase 2 testing is still needed. Our aims are to 1) Use MDR technology and MC to identify individuals at high-risk for MSKI 2) Evaluate whether MDR has diagnostic accuracy superior to MC 3) Develop MDR algorithms that enhance accuracy and enable automation.

**Methods and findings:**

A case control study will compare the movement patterns of 125 ACL reconstruction patients to 125 healthy controls. This study was reviewed and approved by the Pennsylvania State University Human Research Protection Program (HRPP) on May 18, 2022, and the IRB approval number is STUDY00020118. The ACL group is used as a model for a “high risk” population as up to 24% will have a repeat surgery within 2 years. An 8-camera Motion Analysis MC system with Cortex 8 software to collect MC data. Components for the radar technology will be purchased, assembled, and packaged. A micro-doppler signature projection algorithm will determine correct classification of ACL versus healthy control. Our previously tested algorithm for processing the MDR data will be used to identify the two groups. Discrimination, sensitivity and specificity will be calculated to compare the accuracy of MDR to MC in identifying the two groups.

**Conclusions:**

We describe the rationale and methodology of a case-control study using novel MDR technology to detect individuals at high-risk for MSKI. We expect this novel approach to exhibit superior accuracy than the current gold standard. Future translational studies will determine utility in the context of clinical primary care.

## Introduction

### Burden on military and athletes

Non-combat-related musculoskeletal injuries (MSKI) account for 80% of Service Members’ injuries, 60% of limited duty days, and 65% of days in which US Service Members are unable to deploy [1]. In 2014, MSKI accounted for $980 billion in healthcare dollars (5.76% of the annual Gross Domestic Product) [2]. MSKI are the greatest medical threat to Service Members’ health and readiness [3]. In addition, MSKIs result in increased military separation [4], exorbitant medical costs [5], and the development of chronic pain or long-term disability [6]. The impact of MSKI is not unique to the military; during the 2014–2015 National Basketball Association season, it was estimated that MSKI accounted for a loss of $344 million in player salaries [7]. During the 2019–2020 National Football League season, an estimated $521 million was spent on the treatment of MSKI [7]. Service Members and athletes represent younger and more physically fit subpopulations in which MSKI can substantially impair performance and have life-long consequences.

### Preventing musculoskeletal injuries

Primary MSKI risk reduction efforts have shown promise in decreasing MSKI rates; however, current screening methods still lack sufficient accuracy to identify injury risk [8]. The Center for the Intrepid and National Intrepid Center of Excellence have developed sophisticated assessment tools that are available, but require specialized facilities making their use dependent upon proximity to one of these centers. Comprehensive MSKI prediction modeling has potential to identify those at most risk; however, this requires extensive expertise and time, which limits its large-scale implementation [1, 3, 9]. Motion capture (MC) labs provide us with the most objective biomechanical parameters to accurately measure three-dimensional joint and body positions. These labs can be considered the gold standard for accessing those at risk for injury. Unfortunately, MC systems used for measuring whole body activities are large, expensive, have limited portability, and have spatial resolution capabilities limited to approximately what can be seen by the human eye [10].

An alternative approach to human motion analysis is the use of micro-Doppler radar (MDR), which is small, inexpensive, easily portable, and has been proven to distinguish subtle differences in movement patterns not detectable by the human eye [11, 12]. MDR has the ability to detect and evaluate movement at a scale that has never been visually distinguishable to date. In a previous study, MDR predicted when athletes had a 2 cm heel lift in their shoes 100% of the time, reflecting great accuracy in detecting subtle changes in movement patterns [12]. The radar’s accuracy in detecting micro-movements is the premise for using it to screen Service Members or athletes for the risk of MSKI. Identifying those with a high-risk of MSKI will allow limited preventative resources to be directed to the highest-risk population. Ultimately, this approach can decrease injury risk, incidence, and MSKI burden.

Relevant work on the use of MDR for gait analysis includes a study by Seifert et al., in which the researchers validated the feasibility of using Doppler radar to measure clinically relevant gait parameters [13]. The values of five spatiotemporal parameters extracted from radar measurements matched those obtained using MC. Additional data on kinematic parameters showed that measured radar velocities also agreed with the values obtained using MC, reflecting the feasibility of using MDR for gait analysis [13]. Another approach to gait analysis using MDR utilized trunk movement to estimate biomechanical gait parameters instead of the conventional leg-movement-based method [14]. Changes in leg positions relative to the radar make the extraction of ankle-echoes challenging, especially in practical conditions that do not involve walking on a treadmill [15–17]. Saho and colleagues demonstrated that the novel trunk method enables the estimation of the swing and stance times and that the inclusive trunk and leg-based method improves the accuracy of gait estimations [14]. These studies highlight the concept that MDR can be used in a manner similar to how motion capture labs have captured human mechanics previously, but with the advantages of MDR technology.

One high-risk group that has been studied extensively is patients with a history of an anterior cruciate ligament reconstruction (ACLR). Despite many years of research, patients with ACLR have a 6–24% chance of an ACL re-tear or a subsequent knee surgery on either side within 2 years of successful surgical intervention and post-surgical rehabilitation [18]. This could be due to movement deficits that persist beyond the typical rehabilitation period of nine months and are undetectable even by the trained eye [19]. Due to the known risk of re-injury, this population is one that needs further investigation beyond previous measures. Therefore, due to the accuracy of MDR, there may be an opportunity to utilize this technology for evaluating this high-risk population.

The previously completed work has shown that radar technology is an evolving and promising alternative to traditional motion analysis [11, 12]. The purpose of this paper is to describe a case-control study using novel methodology utilizing MDR to detect an at-risk group for MSKI. Our study aims to assess the feasibility of using MDR for identifying populations at risk of MSKI, to determine if MDR is superior to MC, and to advance MDR algorithms to enhance accuracy and prepare for automated data processing. Our hypothesis is that MDR will outperform MC in its ability to identify this high-risk population.

## Methods and materials

### Study design

This is a case-control study using MDR to compare the movement patterns of 125 adults with a history of ACLR to that of 125 healthy controls. This study was reviewed and approved by the Pennsylvania State University Human Research Protection Program (HRPP) on May 18, 2022, and the IRB approval number is STUDY00020118. Eligibility criteria can be found in Table 1.

**Table 1:** Inclusion and exclusion criteria.

	ACL Group	Control Group
**Inclusion Criteria**	Age 18–40	Age 18–40
	History of ACL reconstruction between 9–24 months before recruitment	No history of lower extremity surgery
	Medically cleared to resume normal activities	
**Exclusion Criteria**	Age <18 or >40	Age <18 or >40
	Pregnant or institutionalized	Pregnant or institutionalized
	Current neuromuscular disease	Current neuromuscular disease
	Inability to provide informed consent	Inability to provide informed consent
	Inability to perform study activities	Inability to perform study activities
	Inability to walk or jump without a limp	Inability to walk or jump without a limp
	History of cerebral vascular accident	History of cerebral vascular accident
	History of knee or hip replacement	History of lower extremity surgery

Recruitment will occur through a retrospective review of Electronic Health Records (EHR) to identify patients that have undergone ACLR within the past 9–24 months. These participants will be mailed a recruitment letter explaining the study and providing contact information for our study coordinator. Once the potential participant makes initial contact and shows interest, a link will be sent to their email with a REDCap database eligibility screener [20]. Additional recruitment will occur through a prospective review of the surgical schedule for future ACL surgery candidates or patients that have recently undergone surgery, but have not yet been cleared for full activity. These participants will be provided with study flyers by a member of their medical team, and if interested will contact the study coordinator for potential future recruitment. Participation of all recruited patients is conditional upon successful clearance for full activity by their medical team at the time of participation.

A control cohort will be recruited through study finder (institutional study database), and study recruitment flyers will be distributed throughout the participating institutions. All attempts will be made to match the cases and control by sex, body mass index (BMI), and age.

Interested individuals from both groups will be asked to complete a REDCap eligibility screener and will be directed to a summary explaining the research and electronic consent if found eligible. If consent is given, contact information and basic demographic data will be obtained through REDCap. A study member will then contact eligible and consented individuals to schedule their data collection time.

### Micro-Doppler radar system

The components needed for the assembly of the MDR will be purchased and packaged in a way that makes it easily transported and calibrated, based on previously published parameters for the radar system [11, 12]. The radar will be installed within a MC human performance lab so that the MDR data can be collected simultaneously with the MC data. The antennas for the radar system will be mounted at a height approximately 49 degrees to the participant. Because participants will be performing multiple activities at 0 degrees to the radar from varying distances, measurement distance will not be standardized. The radar itself will be set up outside of the data collection area so as to not disrupt the MC data collection with corresponding cables connected to the antennas and to the computer used for processing and storage of the pre-processed data.

### Motion capture system

The Human Performance Lab consists of 2000 sq. ft. of dedicated space designed for human movement analysis. The lab contains an 8-camera Motion Analysis MC system with Cortex 8 software (Motion Analysis Corp., Rohnert Park, CA) to collect MC data. This system represents the state-of-the-art technology in MC. The MC system cameras is Kestrel and they will be spread over an area of 5 x 3 x 2 meters.

### Data collection

Upon arrival for their data collection session, the participant’s identification number will be checked, and they will fill out the Brief Pain Inventory Short Form, which will serve to identify any confounding pain effects on the testing protocol [21]. To mitigate the effects of possible fluctuations in pain perception, the pain survey will be administered immediately prior to the start of data collection.

Radar Micro-doppler signatures and biomechanical MC data will be simultaneously collected as participants perform a series of functional activities. Combining MC and MDR data collection presents unforeseen challenges. MC techniques are widely known and have developed a level of sophistication that allows for flexibility when positioning equipment in the data collection volume and selection of a marker set. Conversely, MDR is direction-specific in regard to the position of the antennae which in turn dictates the primary plane of action for the experimental task. Since body segment velocities for all three tasks in this experiment are primarily in the sagittal plane it is necessary to align the experimental apparatus with the MDR signal axis (0 deg from center of antennae). Once aligned, the challenge is to select a marker set where the experimental apparatus doesn’t obscure markers. The solution is to place extra tracking markers on the pelvis, forearms (wrists), and head. These markers can then be used as reference points in Visual 3D to create virtual markers, which are then used along with the actual markers to create the skeletal model.

The marker set used to produce the Visual 3D skeletal model (virtual and actual) consists of 34 markers which are used to calculate all joint and segment angles, and their time derivatives. Bilateral lower-body markers consist of medial and lateral toes (2nd metatarsal and little toe), heel (calcaneus), medial and lateral ankle (malleolus), and medial and lateral knee (Tibial condyles). Trunk markers consist of right and left (R&L) anterior superior iliac spine, R&L posterior superior iliac spine, R&L shoulder (acromion), lower neck (C7), mid-trunk (T8), and sternal notch. Bilateral arm markers consist of the medial and lateral elbow (humeral medial and lateral epicondyles) and the wrist (radial and ulnar styloid processes). Head markers consist of the left and right zygomatic arches directly anterior to the inter-aural axis and top of the head.

Data from the ACLR and control groups will be collected by the MDR and MC systems simultaneously by a triggering system that matches the data collection on the time axis prior to the onset of movement. Participants will be asked to perform five trials of each of the following three tasks: Two-legged drop jumps (DJ2) from a low box (30cm), standing from a seated position, i.e., sit-to-stand (STS), and continuous gait on a treadmill. Participants will be asked to wear appropriate clothing and footwear for exercise training. Each task will be demonstrated by a research team member and participants will be given time to practice before data collection begins. Task order will be randomized, and all five trials of each task will be performed as a block. Participants will be given time to rest between practice and trial performances and in between trials during data collection to minimize fatigue effects. Any trial that deviates from the instructed methods will be evaluated and repeated before continuing. The three tasks will be completed in the frontal plane or 0 degrees with respect to the MDR.

The DJ2 task is usually performed without incident in clinically recovered patients during late or post-ACL reconstruction rehabilitation, while the STS and gait are typically applied earlier in the rehabilitation periods for patients who have not fully cleared rehabilitation. The three tasks selected are typical of ACLR rehabilitation, can be performed in any clinic, and cover a range of performance abilities.

### Task 1: Timed Two-Legged drop jumps (DJ2)

Participants will step off a 30 cm high box with their arms held across their chest, landing with both feet simultaneously such that the fall distance is equal to the box height. Participants have to immediately and without hesitation execute a vertical jump (no horizontal translation) upon landing from the box jump. The participant’s return to an upright stationary standing position following the jump marks the end of that trial. Five trials will be performed with 30–60 seconds of rest in between.

### Task 2: Timed Sit-to-Stand (STS)

Participants will be seated on a height-adjustable, armless, flat seat while maintaining a vertical trunk position with their arms held across their chest. Seat height will be adjusted to ensure a 90-degree angle between the thigh and shank with feet flat on the ground. Participants will stand up quickly to reach a vertical standing position. Maintaining the final vertical standing position marks the end of the trial. Five trials with 30–60 seconds of rest in between will be performed.

### Task 3: Gait speed

Preferred gait speed will be estimated by having participants perform three trials of timed walks on a 10-meter measured hallway at their preferred pace. Preferred gait velocity will be estimated as the average velocity (m/s) of the three trials. A single 2.5 minute-gait trial will be performed by participants on a level treadmill at their preferred gait velocity. Only the last 2 minutes of the gait trial will be used for analysis.

### Micro-Doppler signature processing

The process of extracting unique information from MDR begins with the construction of micro-Doppler signatures (MDS). There are various procedures for generating MDS that have been proclaimed as useful in helping discern between physical activities monitored by MDR. The most common approaches extract features from signatures constructed through time-frequency and cadence-frequency diagrams. After obtaining such signatures, activity classification often starts in the form of extracting features from the created signatures. One of the most prominent methods used when performing activity classification with MDR configurations is through the analysis of time-frequency representations of the received MDS. Time-frequency signatures implement the short-time Fourier transform (STFT) for analyzing MDS. After constructing signatures for each activity measured by the MDR, further processing is incorporated into the signatures for extracting unique information. A variety of methods have been employed in extracting features embedded in the signatures. There are two types of feature sets evaluated in this work. One type of feature is deemed “traditional,” being that they are handcrafted from the raw data. The other types of features are those extracted through machine learning techniques, such as dimensionality reduction. More specifically, we propose a dimensionality reduction technique that, as far as we have found, has yet to be implemented in the classification of human gait classification via MDR. We previously developed the micro-Doppler signature processing algorithm (mD-SPA) for processing the MDS to classify specific movement conditions [12]. We will apply the same algorithm to the three performance tasks discussed above in order to distinguish the ACLR group from the control group and determine the accuracy and predictability of the algorithm. This data will be used for comparison to the MC outcome data (outlined below). We will then calculate the sensitivity and specificity of MDR to the MC data to determine whether the participant is in the ACLR group or the control group.

### Motion capture processing

MC data will be analyzed in two ways: 1) traditional MC data analysis will occur using variables extracted from averaged trial data, and 2) MC data will be subjected to the same machine learning algorithms outlined in the MDS processing section. The difference is that there are specific variables for MDS and MC. Extracted MC variables will include: internal rotation of the knee, pronation of the foot, the difference between inter-knee distance, forward displacement of the ACLR hip relative to the uninjured hip, sagittal and frontal hip and knee angular deficits at peak knee flexion angle, sagittal plane hip and knee displacement at peak knee extension angle, sagittal knee and hip displacements during weight acceptance, sagittal knee and hip displacement at midstance, and peak knee flexion and displacement. Data will be analyzed, and movement variables extracted from processed data using Visual 3D (C-Motion, Inc., Boyds, MD) and custom programs written in MatLab (The Mathworks, Inc., Natick, MA).

Traditional MC processing occurs by calculating inverse dynamics using Visual 3D and/or custom MatLab programs. Note that the selected tasks and variables represent measures that are expected to be identifiable using MDR, but not necessarily by MC. It is expected that MC will successfully measure the DJ2s, will be marginally successful at measuring STS, and will be unsuccessful at measuring gait speed on a level treadmill. The data will be processed using typical MC methods. MC data will be cleaned and preprocessed (including filling in missing data) using methods established for software programs Cortex 8 and Visual 3D. Data will be filtered using a 4th order, zero-lag low-pass Butterworth filter using MatLab filtfilt with a 10 Hz cutoff frequency.

The MC data will help us to effectively compare the accuracy of the MC system to that of MDR. Due to their heterogeneity, the two traditional sets of pre-processed data will not be statistically comparable. As such, we will use similar machine learning techniques to process the MC data, so that we are able to determine the accuracy and predictability of MC to correctly classify the ACLR group versus control. Accomplishing this will allow direct comparisons of discrimination, sensitivity and specificity for each of the techniques to successfully predict the control and ACLR groups.

### Sample size justification and data analysis

This sample size calculation justifies our ability to assess the sensitivity and specificity of mD-SPA in its identification of the ACLR (i.e., case) and control groups. A total sample size of 250 (125 ACLR and 125 control) provides 88% power to detect a minimal change of 10% in sensitivity/specificity based on the exact binomial test. This sample size also has 80% power to detect an 8% improvement in prediction accuracy of mD-SPA relative to MC based on McNemar’s test. These calculations assume a minimal sensitivity and specificity of 90% for mD-SPA and that the proportion of discordant pairs of classification between the two measurement systems is 10%. The target significance level is assumed to be two-sided at 0.05 (PASS 2021 v*21*.*02*).

The continuous outcome times for DJ2, STS, and gait speed will be modeled using mixed effects models with person-specific random intercepts. These models will be weighted with the inverse of each person’s probability of belonging in their actual ACLR group, based on their separately modeled propensity for being in that group. The models will also adjust for the explanatory variables in the propensity score model, thereby enjoying the analytic property of being doubly robust. Statistical significance will be justified for multiplicity within the models of DJ2, STS, and gait speed using the standard Benjamini-Hochberg procedure to control for false discovery.

## Discussion

### 1) Feasibility of using MDR

Here we propose a novel methodology using MDR to observe human movement and identify micro-movements indicative of high-risk for MSKI. MDR’s ability to identify subtle differences in movement patterns from the presence or absence of a foam wedge in a shoe’s heel has already been demonstrated in our previous study [12]. This reflects the potential of using MDR for detecting clinically relevant movements that are undetectable by the human eye. Individuals who have undergone ACLR are at high risk for additional surgeries following their initial reconstruction, making them a population at elevated risk. Successfully completion of this protocol will help us to identify this high-risk group with greater accuracy and predictability.

Moving forward, we see significant clinical utility for this technology. MDR technology is highly accurate and requires minimal training to operate. It is also compact, inexpensive, and portable, making it easy to place in a clinical setting or for use in the field. Future aims will determine if retrospective analysis can identify athletes that have incurred injury. Ultimately, we will use these data sets to better understand and refine how MDR identifies individuals at high risk. With this knowledge, we will have information to guide the prospective identification of high-risk individuals for MSKI. These factors may make MDR a potential assessment tool that can have large-scale applications in across a broad spectrum of clinical settings.

### 2) Superiority of MDR to MC

MC is limited by the measurement resolution of the system (typically 1.2–1.5 mm) which, as stated earlier, is on par with visual observation. We have previously shown that MDR can accurately identify subtle changes in movements patterns not detectable by the human eye [12]. Due to its potentially superior measurement sensitivity, MDR represents a viable method for identifying sub-visual risk markers in post-rehabilitation ACL reconstruction patients.

Based on previous work we hypothesize that MDR’s threshold for discriminating subtle movement patterns will be lower than that of MC for squat jumps [12], but the effectiveness in identifying movement pattern changes for other tasks is unknown. We will compare MC with MDR while participants perform drop jumps, sit to stand from a chair, and walk on a treadmill. We hypothesize that MDR will be superior to MC in differentiating these movements in an ACLR group compared with controls. Completion of this aim will provide evidence that MDR is noninferior and likely superior to the current gold standard. This will continue to support the tremendous clinical potential of this inexpensive and portable technology.

Successful completion of this study will provide a sound basis from which to conduct future large-scale prospective clinical trials to expand detection using MDR for specific MSKI injury patterns. This will in turn allow for predictive identification of those injury patterns prior to an actual MSKI. The ability to identify a high-risk MDS pattern is a novel approach that may provide primary prevention to those at high-risk of MSKI. We hypothesize this will in turn allow for targeted focus of limited resources for high-risk populations, in hopes of dramatically reducing patient morbidity, mortality, and associated healthcare costs.

## Methodological limitations and solutions

There are some limitations to using machine learning in the clinical setting. Machine learning will not explain why or how the movements indicate the level of participant risk. Machine learning algorithms create a “black box” phenomenon in which only the input and output data can be seen and not the details of how the processing occurred [7]. For instance, if a participant has significant valgus knee collapse as a risk factor for ACL injury, the algorithm might accurately classify them as high-risk without identifying knee valgus as the root cause of the increased risk. Fortunately, validated injury prevention programs exist [22–24], and the majority are exercise based. In general, the focus has been on improving the quality of athletes’ functional motion through plyometric, agility, and strength training [22–24]. However, universal implementation of such prevention programs is another limitation that the developed mD-SPA algorithm can help overcome [25]. The mD-SPA algorithm could potentially help direct limited preventative resources towards high-risk populations. Ultimately, continuing future data collection will enhance our algorithms by providing the training models with substantially more data to enable further enhancement of their accuracy and significance.

Another limitation is the complexity of MSKI, which is determined by many extrinsic and intrinsic risk factors [26]. Some of these risk factors are: history of injury, previous work restrictions, or lower perceived recovery from injury [9]. Inevitably there may be genetic factors that also play a role that would be of interest as well. Algorithms will need to be strengthened by adding data points taking into consideration the aforementioned risk factors.

A final limitation of MDS processing is its reliance on the impaired resolution of the short-term Fourier transform (STFT). As the duration of data collection increases, the STFT time resolution worsens even as frequency resolution improves. The opposite is also true. However, we were able to overcome this limitation as preliminary work showed that the STFT is sufficiently resolute to illustrate the overall structure of the MDS, even if it is not the optimal tool for subsequent feature extraction and classification.

## Conclusion

We describe the rationale and methodology of a case-control study using novel MDR to detect MSKI and identify individuals in a high-risk group. We expect this novel approach to be accurate and outperform the current gold standard of MC. Future translational studies will determine utility in the context of clinical primary care. Findings from this study may lead to larger prospective trials that will develop a database of micro-Doppler signatures that can identify movement patterns associated with MSKI.
